# Additional Axillary Disease After Neoadjuvant Chemotherapy for Patients with Clinically Node-Negative but Sentinel Node-Positive Disease

**DOI:** 10.1245/s10434-025-18045-7

**Published:** 2025-08-20

**Authors:** S. Jensen, T. F. Tvedskov, T. Bechmann, T. Tramm, M.-B. Jensen, H. B. Rahr, M. D. Lautrup

**Affiliations:** 1 Institute of Regional Health Sciences, University of Southern Denmark, Odense, Denmark; 2 Department of Surgery, University Hospital of Southern Denmark, Vejle, Denmark; 3 University of Copenhagen, Copenhagen, Denmark; 4 Department of Breast Surgery, Gentofte Hospital, Copenhagen, Denmark; 5 Department of Oncology, Regional Hospital West Jutland, Gødstrup, Denmark; 6 Department of Pathology, Aarhus University Hospital, Aarhus, Denmark; 7 Department of Clinical Medicine, Aarhus University, Aarhus, Denmark; 8 Danish Breast Cancer Group (DBCG), Department of Oncology, Copenhagen University Hospital, Copenhagen, Denmark; 9 Department of Plastic and Breast Surgery, Aarhus University Hospital, Aarhus, Denmark

**Keywords:** Breast Cancer, Neoadjuvant Chemotherapy, Axillary metastasis, Isolated tumor cells, Sentinel lymph node dissection, Axillary lymph node dissection

## Abstract

**Background:**

Axillary lymph node dissection (ALND) is associated with considerable risk of arm morbidity. This study aimed to identify risk factors for additional disease found at ALND in patients with a diagnosis of clinically node-negative (cN0) breast cancer who had sentinel lymph node (SLN) metastasis after neoadjuvant chemotherapy (NACT) as a help to identify patients for whom ALND may be safely omitted.

**Methods:**

This retrospective national cohort study analyzed Danish patients with a diagnosis of cT1-3 breast cancer and cN0 disease from October 2016 to November 2022 but a positive SLN biopsy after NACT. Uni- and multivariable logistic regression analyses with stepwise backward elimination were performed to identify factors predictive of additional axillary disease at ALND.

**Results:**

The study identified 346 patients eligible for analyses. Additional disease at ALND was found in 111 patients (32 %), but only 11 % of those with isolated tumor cells in the SLNs had additional axillary disease. It was respectively 22 % and 42 % for micro- and macrometastases. The final multivariable model identified the size of the metastatic deposit in the SLN, the ratio of positive SLNs, extracapsular extension, multifocality, and clinical T-stage as significant predictors of additional axillary disease.

**Conclusion:**

Patients with isolated tumor cells in the sentinel nodes after NACT have a limited risk of additional axillary disease at ALND. However, other factors influence the probability and may be considered in an individualized approach.

**Supplementary Information:**

The online version contains supplementary material available at 10.1245/s10434-025-18045-7.

Sentinel lymph node dissection (SLND) is the standard procedure for staging the axilla in patients with clinically node-negative (cN0) breast cancer. After neoadjuvant chemotherapy (NACT), SLND remains a reliable and safe staging option for patients with cN0 disease, offering a viable alternative to axillary lymph node dissection (ALND),^1–6^ thereby limiting the risks of arm morbidity.^7–10^

Studies have shown comparable risk of axillary recurrence and survival between SLND and ALND in patients with limited disease in the sentinel lymph nodes (SLN) at upfront surgery.^11–15^ These findings have driven a de-escalation of axillary surgery for patients undergoing upfront surgery, as reflected in current guidelines.^16–18^ However, these findings may not fully apply to patients treated with NACT due to variations in tumor subtypes, potential alterations in lymphatic drainage after NACT, and concerns about therapy resistance.^4,6^ As a result, axillary treatment recommended for patients treated with NACT has not undergone the same degree of de-escalation.

The Danish guidelines recommend ALND for all patients with breast cancer and a metastatic SLN after NACT regardless of the size of the metastasic deposit.^18^ Some international guidelines suggest omission of ALND for patients with limited nodal burden after NACT, especially patients undergoing adjuvant radio- or chemotherapy.^16,17^

Determining risk factors for additional axillary disease in patients with a metastatic SLN after NACT is important for identifying patients who may not benefit from ALND and avoiding unnecessary surgery and morbidity.^7–10^ Most studies on this topic are retrospective investigations based on single-institution data with small study populations, including both clinically node-negative and node-positive cases.^19–29^ This study aimed to assess the risk of additional metastases in patients with breast cancer and a positive SLN after NACT despite cN0 status at the time of diagnosis, and to evaluate risk factors for additional axillary disease, with particular attention to the size of the metastatic deposit in the SLN.

## Methods

### Data Source and Data Extraction

Two databases provided information: the Danish Breast Cancer Group (DBCG) database and the Danish Pathology Data Bank (DPDB). The DBCG describes national guidelines for breast cancer diagnosis and treatment.^18^ A prospectively maintained database complements the guidelines, providing information on demographics, pathology, and surgical/oncologic treatment. The DBCG database covers more than 95 % of all breast cancer cases in Denmark.^30^ The DPDB offers detailed records of pathologic analyses for all patients treated in the Danish health care system, with almost 100 % coverage since 1997.^31^ The DPDB was used to supplement pathologic data missing from the DBCG database and provided additional details on extracapsular extension and the Ki67 index. Ethnicity is not routinely recorded in Danish registers and was therefore unavailable for this study. The study followed the Strengthening the Reporting of Observational Studies (STROBE) guidelines.^32^

### Procedure/Danish Guidelines

In Denmark, the standard evaluation of the axilla at diagnosis follows the DBCG guidelines. It is a combination of axillary ultrasound and fine-needle aspiration or core-needle biopsy in case axillary ultrasound shows suspicious axillary lymph nodes. After NACT, all patients with clinically node-negative disease undergo SLND with dual tracer (blue dye and radioactive tracer). Axillary lymph node dissection is recommended to all patients with axillary lymph node metastases of any size after NACT - also in case of isolated tumor cells (ITCs).^18^

### Data Definition and Classification

We defined clinical node-negative disease (cN0) as no detected lymph node metastases on physical examination, imaging, or diagnostic biopsy without malignant tumor cells before NACT. At surgery, all stained and/or radioactive lymph nodes or palpably suspicious nodes were considered SLN. Positive lymph nodes included lymph nodes with macrometastases (MAC > 2 mm), micrometastases (MIC > 0.2 but ≤ 2 mm and/or > 200 cells), or ITCs (MIC ≤ 0.2 mm and/or ≤ 200 ITCs). We included the ratio of positive SLNs to account for potential bias introduced by the number of SLNs removed on the number of positive SLNs.

Estrogen receptor (ER) status was positive if at least 1 % of the tumor cells stained positive. Human epidermal growth factor 2 (HER2) status was positive when immunohistochemistry (IHC) demonstrated overexpression (score 3+) or *in situ* hybridization showed HER2 gene amplification according to current guidelines of the American Society of Clinical Oncology (ASCO) and the College of American Pathologists (CAP).^33^ To align with NACT strategies, we combined ER and HER2 status into three groups (HER2+, ER–/HER2–, and ER+/HER2–).

The NACT regimen included chemotherapy with epirubicin, cyclophosphamide, and either docetaxel or paclitaxel, and in the case of HER2-positive disease, also trastuzumab and pertuzumab. In 2021, DBCG updated the guidelines and included carboplatin for patients with ER–/HER2– breast cancer. We chose a minimum cutoff point of four treatment cycles to allow NACT to have an effect and accepted reduced doses without further restrictions.

### Study Population

**Fig. 1:**
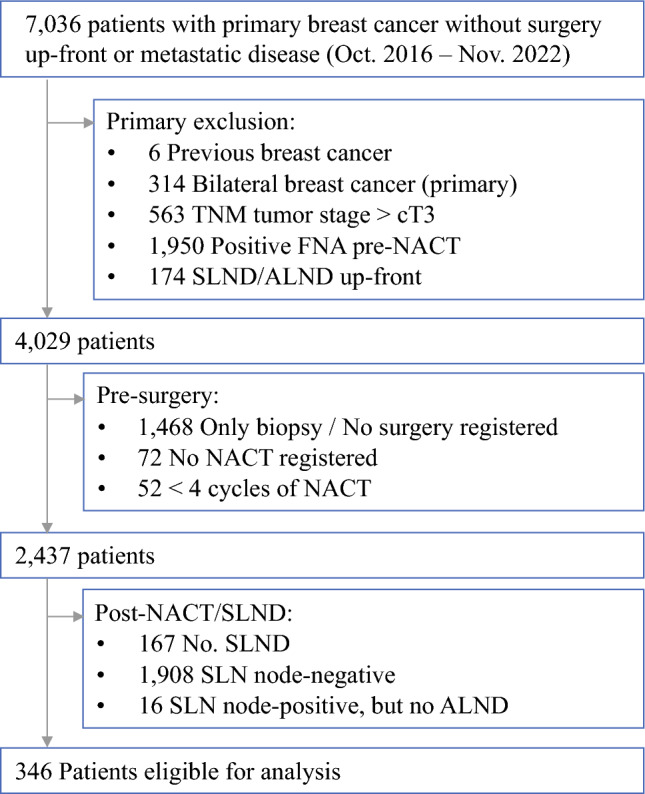
STROBE diagram of the exclusion procress. NACT, neoadjuvant chemotherapy; SLN, sentinel lymph node; SLN node-positive, metastatic deposit of any size; SLND, sentinel lymph node biopsy; ALND, axillary lymph node biopsy

This nationwide study included a prospectively and consecutively collected cohort of patients with cT1-T3 breast cancer diagnosed between 1 October 2016, and 30 November 2022. Patients were identified through the DBCG database. All participants underwent NACT (and HER2-targeted therapy, when indicated). They had no biopsy-detected metastases in the axillary lymph nodes (cN0) at the time of diagnosis, but had metastasis of various extents in the SLN (ypN+ or ypN0i+) after NACT and subsequently underwent ALND. Figure 1 depicts the exclusion process.

### Statistical Analysis

Demographic and clinico-pathologic characteristics are presented as frequencies and percentages for categorical variables. Continuous variables are presented as medians with interquartile ranges. Fisher’s exact test compared the extent of metastatic disease at ALND stratified by the largest metastatic deposit in the SLNs.

Predictive factors for positive lymph nodes at ALND were analyzed by uni- and multivariable logistic regression analyses and reported as odds ratios (ORs) with 95 % confidence intervals (CIs) and *p*-values from the likelihood ratio test, with two-sided *p*-values lower than 0.05 showing significance. To align with previous DBCG studies, the number of positive SLNs was expressed as a ratio of positive to total SLNs, and categorized into two groups in the logistic regression analyses (≤ 2/3 vs > 2/3). Missing values in clinical T-stage and malignancy grade were treated as a separate category. Invasive lobular carcinoma (ILC) was combined with other histologic subtypes, and no/unknown lymphovascular invasion was combined to avoid small numbers.

In creating the logistic regression models, we focused on factors available for the clinicians after SLND and insights from existing literature. For ordinal variables, the lowest category was used as the reference. The exceptions were pathologic tumor size, with ypT1 selected due to limited events in ypT0, and largest metastatic deposit, with MAC selected based on its prevalence. For nominal variables, the predominant group was chosen as the reference.

Model performances were evaluated using the Hosmer-Lemeshow test. Backward stepwise selection determined the final multivariable model, and based on the model, we created a nomogram (Supplementary Information). The area under the curve (AUC) of the receiver operating characteristic (ROC) curve was used to evaluate the predictive performance of the final model after stepwise selection.

Subgroup analysis of the patients with lobular cancers was performed by Fisher’s exact test to account for the difference in tumor biology between lobular and non-lobular breast cancer. The data analyses were performed using StataBE18.

## Results

We identified 346 patients with a clinical tumor stage cT1-3 and ypN+ or ypN0i+ disease after NACT eligible for analyses. Most patients (*n *= 207, 60 %) had MAC in the SLN. Among the remaining patients, 85 (25 %) had MIC and 54 (16 %) had ITC. The clinicopathologic features of our study population are demonstrated in Table 1.

**Table 1:** Patient characteristics and distribution by additional metastases at ALND

	Additional metastases at ALND		
	No (*n *= 235, 67.9 %) *n* (%)	Yes (*n *= 111, 32.1 %) *n* (%)	Total (*n *= 346, 100.0 %) *n* (%)
Age group (years)			
≤ 40	31 (13.2)	15 (13.5)	46 (13.3)
41–60	158 (67.2)	76 (68.5)	234 (67.6)
> 60	46 (19.6)	20 (18.0)	66 (19.1)
Clinical T-stage			
cT1 (< 2 cm)	32 (13.6)	9 (8.1)	41 (11.8)
cT2 (2–5 cm)	172 (73.2)	72 (64.9)	244 (70.5)
cT3 (> 5 cm)	25 (10.6)	24 (21.6)	49 (14.2)
Missing	6 (2.6)	6 (5.4)	12 (3.5)
Histologic type			
IDC	220 (93.6)	98 (88.3)	318 (91.9)
ILC	13 (5.5)	13 (11.7)	26 (7.5)
Other	2 (0.9)	0 (0.0)	2 (0.6)
Immunohistochemical subtype			
ER+/HER2–	158 (67.2)	90 (81.1)	248 (71.7)
ER+/HER2+	47 (20.0)	13 (11.7)	60 (17.3)
ER–/HER2–	25 (10.6)	7 (6.3)	32 (9.2)
ER–/HER2+	5 (2.1)	1 (0.9)	6 (1.7)
Malignancy grade			
I	52 (22.1)	25 (22.5)	77 (22.3)
II	138 (58.7)	64 (57.7)	202 (58.4)
III	33 (14.0)	18 (16.2)	51 (14.7)
Missing	12 (5.1)	4 (3.6)	16 (4.6)
Ki67 index (IQR)^a^	30.0 (15.0–40.0)	25.0 (15.0–50.0)	25.0 (15.0–47.0)
Multifocal cancer			
No	183 (77.9)	72 (64.9)	255 (73.7)
Yes	52 (22.1)	39 (35.1)	91 (26.3)
Pathologic T-stage			
ypT0 (0 mm)	8 (3.4)	3 (2.7)	11 (3.2)
ypT1 (≤ 20 mm)	143 (60.9)	52 (46.8)	195 (56.4)
ypT2 (21–50 mm)	73 (31.1)	44 (39.6)	117 (33.8)
ypT3 (> 50 mm)	11 (4.7)	12 (10.8)	23 (6.6)
Lymphovascular invasion			
No	211 (89.8)	95 (85.6)	306 (88.4)
Yes	22 (9.4)	15 (13.5)	37 (10.7)
Missing	2 (0.9)	1 (0.9)	3 (0.9)
Median no. of SLN (IQR)	2.0 (1.0–3.0)	2.0 (1.0–2.0)	2.0 (1.0–3.0)
Ratio of positive SLNs			
< 1/3	23 (9.8)	0 (0.0)	23 (6.6)
1/3–2/3	111 (47.2)	32 (30.6)	145 (41.9)
> 2/3	101 (43.0)	77 (69.4)	178 (51.4)
Largest metastatic deposit in SLN			
MAC	121 (51.5)	86 (77.5)	207 (59.8)
MIC	66 (28.1)	19 (17.1)	85 (24.6)
ITC	48 (20.4)	6 (5.4)	54 (15.6)
Extracapsular extension in SLN			
No	175 (74.5)	54 (48.6)	229 (66.2)
Yes	60 (25.5)	57 (51.4)	117 (33.8)

ALND, axillary lymph node dissection; IDC, invasive ductal carcinoma; ILC, invasive lobular carcinoma; ER, estrogen receptor; HER2, human epidermal growth factor 2; IQR, interquartile range; SLN, sentinel lymph node; MAC, macrometastases; MIC, micrometastases; ITC, isolated tumor cells

^a^Missing 132 of 347 (38 %); ^b^Ratio of positive SLNs to all SLNs removed

**Fig. 2:**
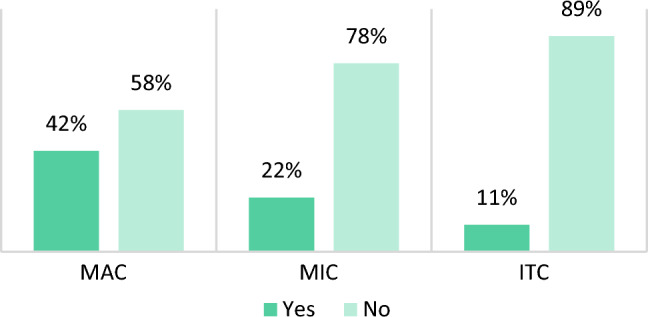
Additional metastases based on the largest metastatic deposit in SLN. SLN, sentinel lymph node; MAC, macrometastases; MIC, micrometastases; ITC, isolated tumor cells

Descriptive analyses showed that a smaller metastatic deposit in the SLN equals a smaller risk of positive lymph nodes at ALND. Only 6 (11 %) of the 54 patients with ITC as the largest metastatic deposit in the SLN had additional metastases at ALND. It was in 19 (22 %) of the 85 patients with MIC and in 86 (42 %) of the 207 patients with MAC, as shown in Table 1 and Fig. 2. The number of additional metastatic lymph nodes at ALND was significantly associated with the size of the metastatic deposit (*p *< 0.01), as demonstrated in Table 2.

**Table 2:** The extent of additional disease at ALND by the largest metastatic deposit in the SLN

No. of positive lymph nodes at ALND	Largest metastatic deposit in SLN		
	MAC (*n *= 207) *n* (%)	MIC (*n *= 85) *n* (%)	ITC (*n *= 54) *n* (%)
0	121 (58.5)	66 (77.7)	48 (88.9)
1	34 (16.4)	14 (16.5)	6 (11.1)
2	16 (7.7)	3 (3.5)	0 (0)
3	13 (6.3)	1 (1.2)	0 (0)
≥ 4	23 (11.1)	1 (1.2)	0 (0)

ALND, axillary lymph node dissection; SLN, sentinel lymph node; MAC, macrometastases; MIC, micrometastases; ITC, isolated tumor cells

Each of the six patients with ITC in the SLN and additional disease at ALND had only one additional metastatic lymph node. In three cases, the additional node contained a MAC, whereas one case contained a MIC, and two cases contained ITC. All six tumors were unifocal invasive ductal carcinomas. Most of the patients were cT2 (83 %) or ER+/HER2− (67 %) and had a positive SLN ratio greater than two thirds (83 %). Extracapsular extension was observed in one case (17 %).

Of the 19 patients with MIC in the SLN and additional disease at ALND, 11 (58 %) had at least one additional node containing a MAC, with a maximum of three MACs found in one case (together with additional nodes containing MIC and ITC). In the remaining eight cases with MIC (42 %), ALND showed a single lymph node with MIC and no further metastases.

Of the 86 patients with MAC in the SLN and additional disease at ALND, 72 (84 %) had at least one additional MAC, with a maximum of 11 MACs found in one patient. In the remaining 14 cases (16 %), ALND showed one to three lymph nodes with MIC as the largest deposit in 11 cases, and one additional lymph node with ITC in 3 cases.

Univariable analyses identified several significant predictors of additional axillary disease, namely, clinical T-stage, tumor subtype, multifocality, pathologic T-stage, presence of extracapsular extension in SLN, ratio of metastatic SLNs, and largest metastatic deposit in SLNs (Table 3). In the multivariable analysis, a smaller metastatic deposit in the SLNs remained significantly associated with a lower risk of additional axillary metastases. Additionally, metastatic involvement of more than two thirds SLNs, extracapsular extension, and multifocality remained a significant predictor of additional axillary metastases. After stepwise backward elimination, clinical T-stage reached statistical significance together with the significant variables in the multivariable analysis (Table 3).

**TABLE 3:** Uni- and multivariable analysis of factors associated with additional axillary disease in Danish patients with cT1–3N0 breast cancer

	Univariable analyses		Multivariable analysis		After backward elimination	
	OR (95 % CI)	*p* Value	OR (95 % CI)	*p* Value	OR (95 % CI)	*p* Value
Age group (years)						
≤ 40	1	0.94				
41–60	0.99 (0.51–1.95)					
> 60	0.90 (0.40–2.02)					
Clinical T-stage						
cT1 (< 2 cm)	1	0.02	1	0.16	1	0.03
cT2 (2–5 cm)	1.48 (0.68–3.27)		1.97 (0.80–4.86)		2.17 (0.91–5.15)	
cT3 (> 5 cm)	3.14 (1.35–8.63)		3.24 (1.07–9.79)		3.91 (1.43–10.72)	
Missing	3.55 (0.92–13.94)		3.62 (0.78–16.92)		4.38 (0.99–19.41)	
Histologic type						
IDC	1	0.10				
ILC and other	1.95 (0.89–4.24)					
Immunohistochemical subtype						
ER+/HER2–	1	0.03	1	0.57		
HER2+	0.47 (0.25–0.90)		0.67 (0.31–1.43)			
ER–/HER2–	0.49 (0.20–1.18)		0.85 (0.32–2.24)			
Malignancy grade						
I	1	0.89				
II	0.96 (0.55–1.69)					
III	1.13 (0.54–2.39)					
Missing	0.69 (0.20–2.37)					
Multifocal cancer						
No	1	0.01	1	0.02	1	0.03
Yes	1.91 (1.16–3.13)		1.94 (1.1–3.41		1.85 (1.07–3.22)	
Pathologic T stage						
ypT0 (0 mm)	1.03 (0.26–4.03)		3.23 (0.69–15.16)			
ypT1 (≤ 20 mm)	1	0.04	1	0.55		
ypT2 (21–50 mm)	1.66 (1.01–2.71)		1.12 (0.64–1.98)			
ypT3 (> 50 mm)	3.00 (1.25–7.21)		1.22 (0.42–3.52)			
Lymphovascular invasion						
No	1	0.25				
Yes	1.51 (0.75–3.04)					
Ratio of positive SLN^**a**^						
≤ 2/3	1	< 0.001	1	< 0.001	1	< 0.001
> 2/3	3.00 (1.86–4.85)		2.74 (1.63–4.59)		2.76 (1.65–4.60)	
Largest metastatic deposit in SLN						
MAC	1	< 0.001	1	0.02	1	0.01
MIC	0.41 (0.23–0.72)		0.65 (0.34–1.28)		0.64 (0.33–1.23)	
ITC	0.18 (0.07–0.43)		0.27 (0.10–0.71)		0.27 (0.11–0.71)	
Extracapsular extension in SLN						
No	1	< 0.001	1	0.01	1	0.01
Yes	3.01 (1.92–4.94)		2.17 (1.24–3.82)		2.17 (1.25–3.77)	

OR, odds ratio; CI, confidence interval; IDC, invasive ductal carcinoma; ILC, invasive lobular carcinoma; ER, estrogen receptor; HER2, human epidermal growth factor 2; SLN, sentinel lymph node; MAC, macrometastases; MIC, micrometastases; ITC, isolated tumor cells;

^a^Ratio of positive SLNs to all SLNs

Using the logistic regression model we estimated the predicted probabilities of additional axillary disease based on the three strongest predictors (largest metastatic size, clinical Tstage, and ratio of positive SLNs). For the patients with ITC in the SLN and no additional risk factors, the predicted probability was 0.02 (95 % CI, − 0.01 to 0.05). Among the patients with ITC, cT2-3, and no additional risk factors, the probability increased to 0.06 (95 % CI, 0.01–0.11). If instead the ratio of positive SLN exceeded two thirds in the patients with ITC and cT1, the probability was 0.06 (95 % CI, − 0.01 to 0.16). When both cT2-3 and a positive SLN ratio greater than two thirds were present, the probability increased to 0.14 (95 % CI, 0.03–0.26). The wide confidence intervals reflect the small number of patients in some subgroup.

**Fig. 3:**
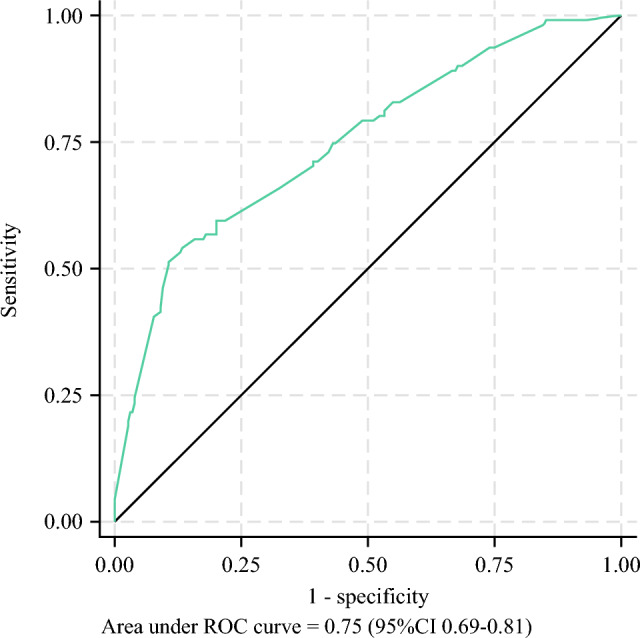
Area under the curve of the final model after backward selection

For the patients with MIC in the SLN, the predicted probabilities were 0.05 (95 % CI, 0.01–0.11) with no additional risk factors, 0.13 (95 % CI, 0.06–0.20) with a cT2-3, 0.14 (95 % CI, 0.02–0.26) with a positive SLN ratio higher than two thirds, and 0.28 (95 % CI, 0.17–0.41) if all three risk factors were present. For the patients with MAC in the SLN, the corresponding probabilities were 0.08 (95 % CI, 0.01–0.16), 0.18 (95 % CI, 0.10–0.27), 0.21 (95 % CI, 0.05–0.36), and 0.40 (95 % CI, 0.27–0.51). If all risk factors were present, the predicted probability was 0.63 (95 % CI, 0.43–0.84). The predicted probabilities of additional axillary disease based on the model are illustrated in a nomogram (Supplementary Information). Evaluation of the model’s discriminative ability resulted in an AUC of 0.75 (95 % CI, 0.69–0.81), depicted in Fig. 3.

Subgroup analysis of the patients with ILC was limited by the small number of patients with ILC (26 patients), half of whom had additional metastases at ALND. As in the overall cohort, larger metastatic deposit was associated with a higher risk of additional metastases. Neither of the two patients with ITC had additional metastases. Among the six patients with MIC, two (33 %) had additional metastases, with one patient having two additional MACs. Among the patients with MAC, 11 (61 %) of 18 patients had additional metastatic lymph nodes, and 8 (44 %) patients had three or more additional metastatic lymph nodes at ALND, indicating a higher nodal burden than in the overall cohort. However, the association was not significant in the lobular subgroup. Fisher’s exact test showed a positive SLN ratio greater than two thirds (*p *= 0,002) and extracapsular extension (*p* = 0,004) as significant predictors of additional axillary disease at ALND for patients with lobular breast cancer.

## Discussion

To our knowledge, this is the largest study to date investigating additional metastatic disease in the axilla among cN0 patients with SLN metastases after NACT. The study highlights that patients with MAC in the SLN are the main contributors to the considerable rate of additional axillary metastases found at ALND (32 %) among patients without detected axillary node involvement before NACT. The risk of additional axillary disease decreases with smaller metastatic deposits. Only 11 % of the patients with ITC, 22 % of the patients with MIC, but 42 % of the patients with MAC had additional axillary disease at ALND. A larger metastatic deposit was also associated with a greater number of additional metastatic lymph nodes at ALND.

Notably, the low percentage of additional disease in patients with ITC compares with the false-negative rate of SLND found to be 7 % to 11 % in clinically node-negative breast cancer patients after NACT.^3–6^ This adds to the question of the benefit of ALND for patients with ITC who receive adjuvant locoregional radiotherapy.

### Risk Factors for Residiual Disease After NACT

The small number of patients with ITC and additional metastases at ALND often results in either excluding patients with ITC or combining ITC and MIC, limiting information on patients with ITC as the largest metastatic deposit.^20,21,28^ A large retrospective multicenter study by Montagna et al.^23^ focused exclusively on the nodal burden and oncologic outcome for patients with ITC in SLN after NACT. Of 583 patients in their study, 150 were cN0, and 30 of these patients underwent ALND. In the overall population, they found additional metastases in 30 % of the patients, but only 5 % had additional MAC at ALND. Some centers used IHC to examine the lymph nodes, which may explain the higher rate of ITC detection in additional lymph nodes at ALND.^26^ In Denmark, national guidelines set minimum requirements for pathologic examination,^18^ but slicing protocols, number of levels examined, and use of IHC vary between institutions. However, IHC examination is not routine in ALND.

Other studies have investigated risk factors for additional axillary disease in patients with breast cancer and SLN metastases after NACT. Proposed risk factors include the extent of the metastatic deposit in the SLNs, extracapsular extension, and number of positive and negative SLNs. Additional factors such as age, tumor size (before and after NACT), multifocality, and biologic characteristics such as ER/HER2– status and lymphovascular invasion also have been reported.^19–29^ Our multivariable analyses confirmed that the size of the largest metastatic deposit in SLN, the ratio of positive SLNs, extracapsular extension, cT-stage, and multifocality were associated with additional axillary disease.

Most previous studies on risk factors for non-sentinel node metastases after NACT included both patients with cN0 and those with cN+ disease.^19–27^ However, the accuracy of SLND may differ between these groups,^1,3–5^ and in Denmark, targeted axillary dissection is standard for patients with cN+ disease.^18^ To our knowledge, only the studies by Cebrecos et al.^28^ and So et al.^29^ focused exclusively on patients with cN0 disease. As in our study, Cebrecos et al.^28^ identified size of the metastatic deposit in the SLN as a predictor.

The association between a higher ratio of positive SLNs and an increased risk of additional axillary disease observed in our study corresponds with the literature.^19,25^ Although no significant difference was found across immunohistochemical subtypes, the ER+/HER2– subtype had the highest odds ratio of additional axillary disease in the univariable analyses. This aligns with findings from other studies, in which immunohistochemical subtype was a significant factor in predicting additional axillary disease.^20,21,27,28^ In our study, patients with missing cT-stage had a high odds ratio (and a wide 95 % confidence interval) of additional axillary disease. This may reflect technical difficulties measuring the tumor size when the size exceeded the ultrasound field of view.

Our model achieved an AUC of 0.75 (95 % CI, 0.69–0.81), indicating fair discriminatory ability. The limited number of events in some subgroups, together with our focus on readily available variables to enhance clinical applicability, may have influenced the AUC. However, by prioritizing simple and widely accessible parameters, we aimed to create a practical model easily translated into various clinical settings. This focus on clinical applicability may have come at the expense of a higher predictive accuracy because incorporating more complex variables could potentially improve the AUC. Differences in inclusion criteria, variables, and statistical methods make direct comparison of AUC with other studies challenging,^24–28^ but our model’s AUC of 0.75 falls within a comparable range.

### Influence on Adjuvant Treatment

Our study challenges the necessity of ALND for patients with cN0 breast cancer before NACT who only have ITC in the SLN afterward, given the minimal risk of additional metastatic disease at ALND. The study by Montagna et al.^23^ omitted ALND for 401 of 583 patients without a significant difference in recurrence after a median follow-up period of 3.2 years, which questions the prognostic benefit of ALND for patients with ITC in the sentinel node after NACT.^23^

The Danish guidelines recommend locoregional radiotherapy for all cases of metastatic disease in the axillary lymph nodes after NACT, including ITC. According to international guidelines, locoregional therapy can often be omitted in case of only ITC.^16,17^ The RAPCHEM study found it safe to omit locoregional therapy for patients with limited nodal disease at ALND after NACT based on the locoregional recurrence rate.^35^ This suggests a simultaneous de-escalation of radiotherapy and surgical treatment of the axilla. Nevertheless, it may be more beneficial to prioritize de-escalation of high-risk axillary surgery first, replacing it with locoregional radiotherapy, which carries a lower risk of arm morbidity.^8–10^ Adjuvant chemotherapy, HER2-targeted therapy, immune checkpoint inhibitor and/or endocrine therapy may be administered based on extent of additional disease (breast and axilla), histologic type, and immunohistochemical subtype.^16,17^ According to the NeoSTEEP criteria, only ypN0 qualifies as an axillary pathologic complete response.^36^ Therefore, none of the patients in our study achieved a complete pathologic response, meaning that all could have been candidates for additional treatment regardless of the ALND result.

## Study Strengths and Limitations

A key strength of this study was its use of high-quality national registries with prospectively and consecutively collected real-world data. Limitations included the relatively small number of events among the patients with ITC (and MIC), especially in the lobular subgroup and observational study design. The proposed nomogram (Supplementary Information) has not been validated in an independent dataset, which limits immediate clinical applicability. Additionally, compliance with national guidelines (although not usually problematic) means that it is not possible to investigate the impact of omitting ALND on recurrence and survival parameters.

## Future Directions

The lack of clear guidelines and evidence supporting the prognostic benefit of ALND for patients with ITC after NACT combined with the risks associated with ALND has led to a growing tendency to avoid ALND, consistent with the general de-escalation of axillary treatment. Prospective studies comparing ALND with axillary radiotherapy for patients treated with NACT in terms of recurrence and survival are ongoing, but most are analyzing clinically node-positive patients, and not all include ITC.^37–40^ The ADARNAT trial includes patients with cN0 breast cancer and positive SLN (and ITC), but endocrine therapy can be part of the neoadjuvant treatment.^41^ Results from these studies in combination with the findings from our study may help determine whether locoregional radiotherapy could replace ALND for patients with ITC in the SLNs after NACT.

## Conclusion

Patients with cN0 breast cancer and ITC in the SLNs after NACT have a minimal risk of additional axillary disease. An individualized approach might be warranted for the few cases with many other risk factors. Assessing the risk is relevant when additional axillary treatment strategies such as ALND and/or locoregional radiotherapy are evaluated.

## Supplementary Information

Below is the link to the electronic supplementary material.

Supplementary file1 (DOCX 85 KB)

## Data Availability

The dataset generated and/or analyzed during the current study is not publicly available due to restrictions by the Danish General Protection Regulation. The data are available from DBCG and DPDB. Metadata are available from the corresponding author upon reasonable request and with permission from DBCG and DPDB.

## References

[1] Kahler-Ribeiro-Fontana S, Pagan E, Magnoni F, et al. Long-term standard sentinel node biopsy after neoadjuvant treatment in breast cancer: a single-institution ten-year follow-up. *Eur J Surg Oncol*. 2021;47:804–12. 10.1016/j.ejso.2020.10.014. 33092968 10.1016/j.ejso.2020.10.014

[2] Hunt KK, Yi M, Mittendorf EA, et al. Sentinel lymph node surgery after neoadjuvant chemotherapy is accurate and reduces the need for axillary dissection in breast cancer patients. *Ann Surg*. 2009;250:558–66. 10.1097/SLA.0b013e3181b8fd5e. 19730235 10.1097/SLA.0b013e3181b8fd5e

[3] Classe JM, Loaec C, Gimbergues P, et al. Sentinel lymph node biopsy without axillary lymphadenectomy after neoadjuvant chemotherapy is accurate and safe for selected patients: the GANEA 2 study. *Breast Cancer Res Treat*. 2019;173:343–52. 10.1007/s10549-018-5004-7. 30343457 10.1007/s10549-018-5004-7

[4] van Deurzen CHM, Vriens BEPJ, Tjan-Heijnen VCG, et al. Accuracy of sentinel node biopsy after neoadjuvant chemotherapy in breast cancer patients: a systematic review. *Eur J Cancer*. 2009;45:3124–30. 10.1016/j.ejca.2009.08.001. 19716287 10.1016/j.ejca.2009.08.001

[5] Shirzadi A, Mahmoodzadeh H, Qorbani M. Assessment of sentinel lymph node biopsy after neoadjuvant chemotherapy for breast cancer in two subgroups: initially node-negative and node-positive converted to node-negative: a systemic review and meta-analysis. *J Res Med Sci*. 2019;24:18. 10.4103/jrms.JRMS_127_18. 30988686 10.4103/jrms.JRMS_127_18 PMC6421883

[6] Mamounas EP, Brown A, Anderson S, et al. Sentinel node biopsy after neoadjuvant chemotherapy in breast cancer: results from national surgical adjuvant breast and bowel project protocol B-27. *J Clin Oncol*. 2005;23:2694–702. 10.1200/JCO.2005.05.188. 15837984 10.1200/JCO.2005.05.188

[7] DiSipio T, Rye S, Newman B, Hayes S. Incidence of unilateral arm lymphoedema after breast cancer: a systematic review and meta-analysis. *Lancet Oncol*. 2013;14:500–15. 10.1016/S1470-2045(13)70076-7. 23540561 10.1016/S1470-2045(13)70076-7

[8] Armer JM, Ballman KV, McCall L, et al. Factors associated with lymphedema in women with node-positive breast cancer treated with neoadjuvant chemotherapy and axillary dissection. *JAMA Surg*. 2019;154:800–9. 10.1001/jamasurg.2019.1742. 31314062 10.1001/jamasurg.2019.1742 PMC6647005

[9] Nguyen TT, Hoskin TL, Habermann EB, Cheville AL, Boughey JC. Breast cancer-related lymphedema risk is related to multidisciplinary treatment and not surgery alone: results from a large cohort study. *Ann Surg Oncol*. 2017;24:2972–80. 10.1245/s10434-017-5960-x. 28766228 10.1245/s10434-017-5960-x PMC5737818

[10] Naoum GE, Roberts S, Brunelle CL, et al. Quantifying the impact of axillary surgery and nodal irradiation on breast cancer-related lymphedema and local tumor control: long-term results from a prospective screening trial. *J Clin Oncol*. 2020;38:3430–8. 10.1200/JCO.20.00459. 32730184 10.1200/JCO.20.00459 PMC7527159

[11] Tinterri C, Canavese G, Gatzemeier W, et al. Sentinel lymph node biopsy versus axillary lymph node dissection in breast cancer patients undergoing mastectomy with one to two metastatic sentinel lymph nodes: sub-analysis of the SINODAR-ONE multicentre randomized clinical trial and reopening of enrolment. *Br J Surg*. 2023;110:1143–52. 10.1093/bjs/znad215. 37471574 10.1093/bjs/znad215 PMC10492188

[12] Houvenaeghel G, de Nonneville A, Chopin N, et al. The need to tailor the omission of axillary lymph node dissection to patients with good prognosis and sentinel node micro-metastases. *Cancer Med*. 2023;12:4023–32. 10.1002/cam4.5257. 36127853 10.1002/cam4.5257 PMC9972015

[13] Galimberti V, Cole BF, Viale G, et al. Axillary dissection versus no axillary dissection in patients with breast cancer and sentinel-node micrometastases (IBCSG 23–01): 10-year follow-up of a randomised, controlled phase 3 trial. *Lancet Oncol*. 2018;19:1385–93. 10.1016/S1470-2045(18)30380-2. 30196031 10.1016/S1470-2045(18)30380-2

[14] Giuliano AE, Ballman KV, McCall L, et al. Effect of axillary dissection vs no axillary dissection on 10-year overall survival among women with invasive breast cancer and sentinel node metastasis: the ACOSOG Z0011 (Alliance) randomized clinical trial. *JAMA*. 2017;318:918–26. 10.1001/jama.2017.11470. 28898379 10.1001/jama.2017.11470 PMC5672806

[15] de Boniface J, Filtenborg Tvedskov T, Rydén L, et al. Omitting axillary dissection in breast cancer with sentinel-node metastases. *N Eng J Med*. 2024;390:1163–75. 10.1056/NEJMoa2313487. 10.1056/NEJMoa2313487 38598571

[16] Loibl S, André F, Bachelot T, et al. Early breast cancer: ESMO clinical practice guideline for diagnosis, treatment, and follow-up. *Ann Oncol*. 2024;35:159–82. 10.1016/j.annonc.2023.11.016. 38101773 10.1016/j.annonc.2023.11.016

[17] Rashmi Kumar N, Schonfeld R, Gradishar WJ, et al. *NCCN Guidelines Version 4.2024 Breast Cancer*. Retrieved 2024 at https://www.nccn.org/.

[18] DMCG kliniske retningsliner for brystkræft (DBCG). Retrieved 25 April 2025 at https://www.dmcg.dk/Kliniske-retningslinjer/kliniske-retningslinjer-opdelt-paa-dmcg/brystcancer/.

[19] Aragón-Sánchez S, Sánchez-Bayona R, López-Marín L, et al. De-escalating axillary management after neoadjuvant chemotherapy in breast cancer: the ratio of positive sentinel lymph nodes matters. *Surg Oncol*. 2024;54:102062. 10.1016/j.suronc.2024.102062. 38531288 10.1016/j.suronc.2024.102062

[20] Johnson NC, Kornfeld H, Gonzalez L, et al. Factors associated with additional axillary disease in patients with positive sentinel lymph nodes after neoadjuvant chemotherapy for breast cancer. *Am Surg*. 2024;90:2614–21. 10.1177/00031348241248813. 38716696 10.1177/00031348241248813

[21] Sanders SB, Hoskin TL, Stafford AP, Boughey JC. Factors influencing non-sentinel lymph node involvement in patients with positive sentinel lymph node(s) after neoadjuvant chemotherapy for breast cancer. *Ann Surg Oncol*. 2022;29:7769–78. 10.1245/s10434-022-12064-4. 35834142 10.1245/s10434-022-12064-4

[22] Moo TA, Pawloski KR, Flynn J, et al. Is residual nodal disease at axillary dissection associated with tumor subtype in patients with low-volume sentinel node metastasis after neoadjuvant chemotherapy? *Ann Surg Oncol*. 2021;28:6044–50. 10.1245/s10434-021-09910-2. 33876362 10.1245/s10434-021-09910-2 PMC10224770

[23] Montagna G, Laws A, Ferrucci M, et al. Nodal Burden and oncologic outcomes in patients with residual isolated tumor cells after neoadjuvant chemotherapy (ypN0i+): the OPBC-05/ICARO study. *J Clin Oncol*. 2024. 10.1200/JCO.24.01052. 39509672 10.1200/JCO.24.01052 PMC11856002

[24] Leonardi MC, Arrobbio C, Gandini S, et al. Predictors of positive axillary non-sentinel lymph nodes in breast cancer patients with positive sentinel lymph node biopsy after neoadjuvant systemic therapy. *Radiother Oncol*. 2021;163:128–35. 10.1016/j.radonc.2021.08.013. 34461184 10.1016/j.radonc.2021.08.013

[25] Tang X, Feng Y, Zhao W, Liu R, Chen N. Prediction of non-sentinel lymph node metastases in T1–2 sentinel lymph node-positive breast cancer patients undergoing mastectomy following neoadjuvant therapy. *World J Surg Oncol*. 2024;22:258. 10.1186/s12957-024-03537-9. 39342230 10.1186/s12957-024-03537-9 PMC11439197

[26] Jeruss JS, Newman LA, Ayers GD, et al. Factors predicting additional disease in the axilla in patients with positive sentinel lymph nodes after neoadjuvant chemotherapy. *Cancer*. 2008;112:2646–54. 10.1002/cncr.23481. 18442039 10.1002/cncr.23481 PMC4365777

[27] Barron AU, Hoskin TL, Boughey JC. Predicting non-sentinel lymph node metastases in patients with a positive sentinel lymph node after neoadjuvant chemotherapy. *Ann Surg Oncol*. 2018;25:2867–74. 10.1245/s10434-018-6578-3. 29956095 10.1245/s10434-018-6578-3

[28] Cebrecos I, Mension E, Alonso I, et al. Nonsentinel axillary lymph node status in clinically node-negative early breast cancer after primary systemic therapy and positive sentinel lymph node: a predictive model proposal. *Ann Surg Oncol*. 2023;30:4657–68. 10.1245/s10434-023-13231-x. 36809608 10.1245/s10434-023-13231-x PMC10319670

[29] So A, Yi M, Simons JM, et al. Significance of residual nodal disease in clinically node-negative breast cancer after neoadjuvant chemotherapy. *Ann Surg Oncol*. 2024. 10.1245/s10434-024-16382-7. 39441324 10.1245/s10434-024-16382-7 PMC11710993

[30] Christiansen P, Ejlertsen B, Jensen MB, Mouridsen H. Danish breast cancer cooperative group. *Clin Epidemiol*. 2016;8:445–9. 10.2147/CLEP.S99457. 27822082 10.2147/CLEP.S99457 PMC5094574

[31] Bjerregaard B, Larsen OB. The Danish pathology register. *Scand J Public Health*. 2011;39(7 Suppl):72–4. 10.1177/1403494810393563. 21775357 10.1177/1403494810393563

[32] Von Elm E, Altman DG, Egger M, Pocock SJ, Gøtzsche PC, Vandenbroucke JP. The Strengthening the reporting of observational studies in epidemiology (STROBE) statement: guidelines for reporting observational studies. *Epidemiology*. 2007;18:800–4. 10.1097/EDE.0b013e3181577654. 18049194 10.1097/EDE.0b013e3181577654

[33] Wolff AC, Somerfield MR, Dowsett M, et al. Human epidermal growth factor receptor 2 testing in breast cancer American Society of clinical oncology-college of American Pathologists guideline update. *Arch Pathol Lab Med*. 2023;147:993–1000. 10.5858/arpa.2023-0950-SA. 37303228 10.5858/arpa.2023-0950-SA

[34] Munck F, Jensen MB, Vejborg I, et al. Residual axillary metastases in node-positive breast cancer patients after neoadjuvant treatment: a register-based study. *Ann Surg Oncol*. 2024;31:5157–67. 10.1245/s10434-024-15354-1. 38704502 10.1245/s10434-024-15354-1 PMC11236906

[35] de Wild SR, de Munck L, Simons JM, et al. De-escalation of radiotherapy after primary chemotherapy in cT1–2N1 breast cancer (RAPCHEM; BOOG 2010–03): 5-year follow-up results of a Dutch, prospective, registry study. *Lancet Oncol*. 2022;23:1201–10. 10.1016/S1470-2045(22)00482-X. 35952707 10.1016/S1470-2045(22)00482-X

[36] Litton JK, Regan MM, Pusztai L, et al. Standardized definitions for efficacy end points in neoadjuvant breast cancer clinical trials: NeoSTEEP. *J Clin Oncol*. 2023;41:4433–42. 10.1200/JCO.23.00435. 37433103 10.1200/JCO.23.00435 PMC10522109

[37] A Randomized Phase III Clinical Trial Evaluating Post-Mastectomy Chestwall and Regional Nodal XRT and Post-Lumpectomy Regional Nodal XRT in Patients With Positive Axillary Nodes Before Neoadjuvant Chemotherapy Who Convert to Pathologically Negative Axillary Nodes After Neoadjuvant Chemotherapy. Retrieved 25 April 2025 at https://www.clinicaltrials.gov/study/NCT01872975.

[38] Tailored Axillary Surgery With or Without Axillary Lymph Node Dissection Followed by Radiotherapy in Patients With Clinically Node-positive Breast Cancer (TAXIS): A Multicenter Randomized Phase III Trial (OPBC-03/ SAKK 23/16 /IBCSG 57-18/ABCSG-53/GBG-101). Retrieved 25 April 2025 at https://clinicaltrials.gov/study/NCT03513614. 10.1186/s13063-018-3021-9 PMC6278139 30514362

[39] A Randomized Phase III Trial Comparing Axillary Lymph Node Dissection to Axillary Radiation in Breast Cancer Patients (cT1–3 N1) Who Have Positive Sentinel Lymph Node Disease After Neoadjuvant Chemotherapy. Retrieved 25 April 2025 at https://clinicaltrials.gov/study/NCT01901094.

[40] Mamounas E, Bandos H, White J, et al. Abstract GS02-07: loco-regional irradiation in patients with biopsy-proven axillary node involvement at presentation who become pathologically node-negative after neoadjuvant chemotherapy: primary outcomes of NRG Oncology/NSABP B-51/RTOG 1304. *Cancer Res*. 2024;84:02–7. 10.1158/1538-7445.SABCS23-GS02-07.

[41] Garcia-Tejedor A, Ortega-Exposito C, Salinas S, et al. Axillary lymph node dissection versus radiotherapy in breast cancer with positive sentinel nodes after neoadjuvant therapy (ADARNAT trial). *Front Oncol.* 2023;13. 10.3389/fonc.2023.1184021. 10.3389/fonc.2023.1184021 PMC10446877 37621686

