# Supplementary figures and images for: Additional Axillary Disease After Neoadjuvant Chemotherapy for Patients with Clinically Node-Negative but Sentinel Node-Positive Disease
Source: Ann Surg Oncol. 2025 Aug 20;32(13):9807–16. doi: 10.1245/s10434-025-18045-7 (PMC12589200; doi:10.1245/s10434-025-18045-7)

Supplemental: Nomogram with Predicted Probability of Additional Axillary Disease


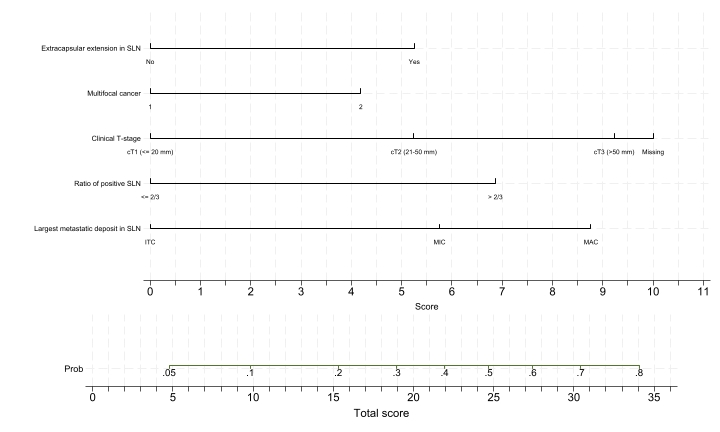

Supplement: Supplementary file 1 — Supplementary file1 (DOCX 85 KB) [file 10434_2025_18045_MOESM1_ESM.docx]
